# Supplementary material for: Dengue virus susceptibility in Aedes aegypti linked to natural cytochrome P450 promoter variants
Source: Nat Commun. 2025 Aug 12;16:7468. doi: 10.1038/s41467-025-62693-y (PMC12343897; doi:10.1038/s41467-025-62693-y)
Supplement: Supplementary file 1 — Supplementary Information [file 41467_2025_62693_MOESM1_ESM.pdf]

Supplementary Information

Dengue virus susceptibility in *Aedes aegypti* linked to natural cytochrome P450 promoter variants

Sarah H. Merkling, Elodie Couderc, Anna B. Crist, Stéphanie Dabo, Josquin Daron, Natapong Jupatanakul, Myriam Burckbuchler, Thomas Vial, Odile Sismeiro, Rachel Legendre, Adrien Pain, Hugo Varet, Davy Jiolle, Diego Ayala, Christophe Paupy, Eric Marois, Louis Lambrechts

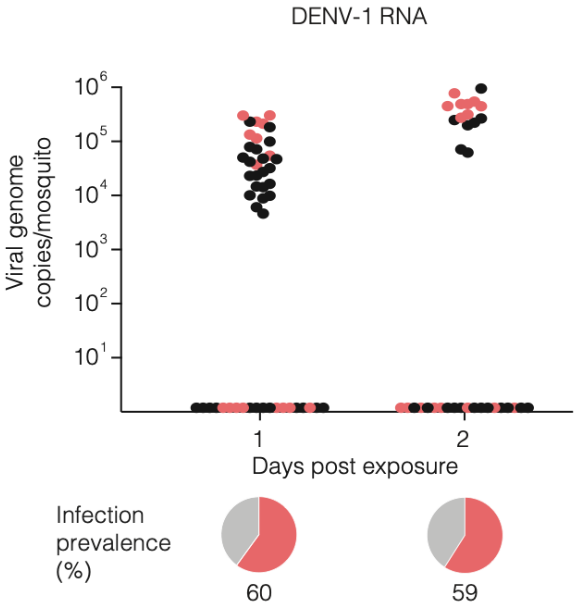

Figure S1. Samples selected for transcriptomic analysis (related to Fig. 1). The figure shows viral RNA levels and infection prevalence of individual mosquito midguts collected 1 or 2 days after an infectious bloodmeal containing 5×10^6 focus-forming units (FFU)/ml of DENV-1. The graph shows the abundance of viral RNA, and the pie charts below represent the proportion of positive individuals. To maximize phenotypic contrast between resistant and susceptible individuals, midguts with the lowest and highest viral RNA levels, respectively, were selected for RNA sequencing and differential gene expression analysis. The selected samples are shown in pink and the non-selected samples are shown in black. The proportion of positive samples on day 1 post infectious bloodmeal (60%) is lower than in Fig. 1c (95%) due to a small difference in viral RNA dynamics between the experiments. Source data are provided as a Source Data file.

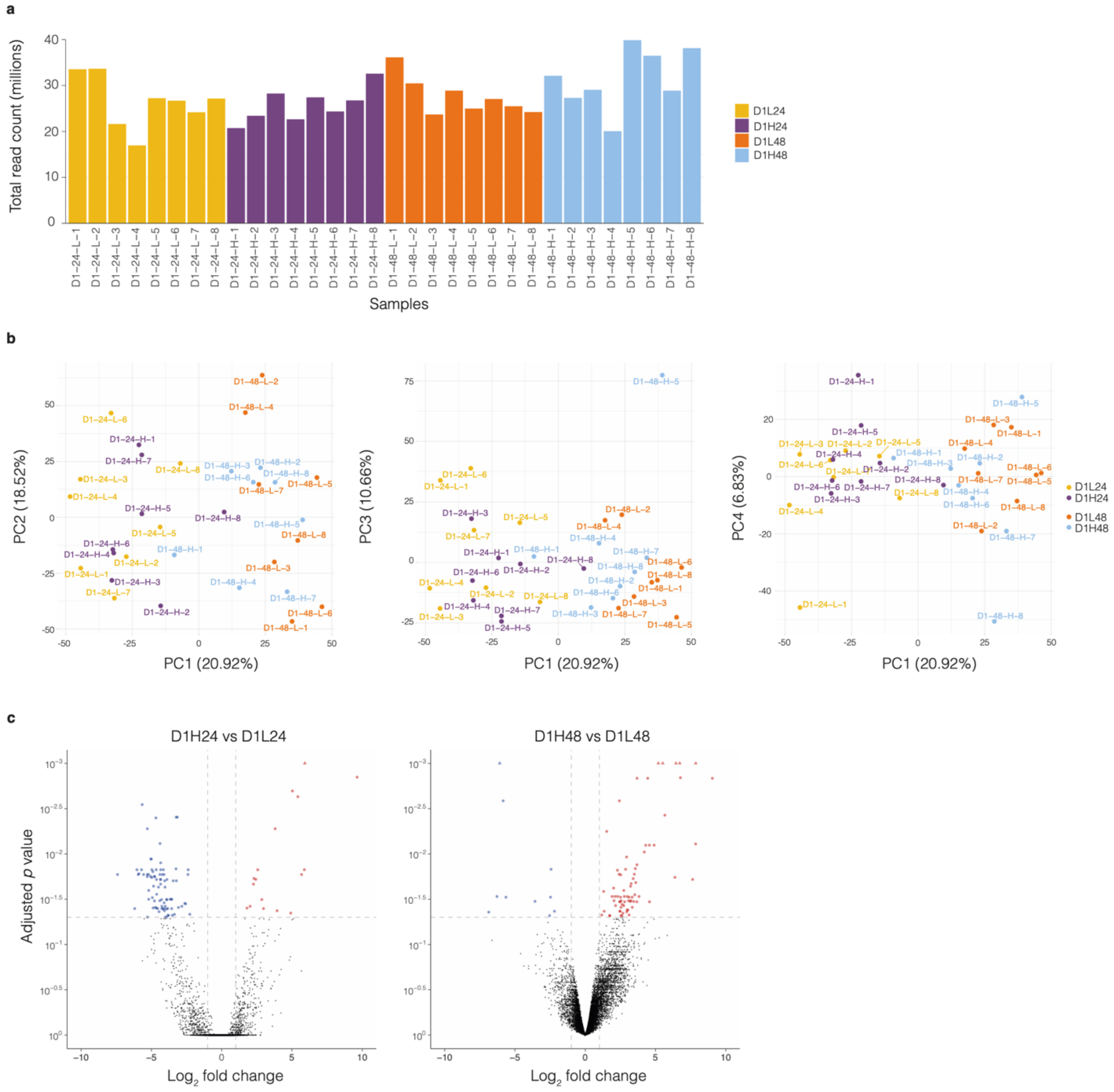

Figure S2. Overview of the RNA-seq dataset (related to Fig. 1). Total read counts per sample (**a**), principal component analysis (PCA) (**b**), and volcano plots (**c**) are shown. In (**a**), the RNA-seq samples are labelled according to four conditions (D1L24, D1H24, D1L48, D1H48) corresponding to 24 and 48 hours post DENV-1 exposure in uninfected (L) and infected (H) mosquitoes. The 8 biological replicates of each condition are labelled as D1-24-L-X, D1-24-H-X, D1-48-L-X, D1-48-H-X, where X is the replicate number. In (**b**), PCA was performed using $\log_{10}$-transformed (normalized) read counts per million. In (**c**), differentially expressed genes between uninfected (L) and infected (H) mosquitoes 24 hours (left) and 48 hours (right) post DENV-1 exposure are shown. Genes with an adjusted *p* value <0.05 and $\log_2$-transformed fold change >1 or <-1 are highlighted in red and blue, respectively.

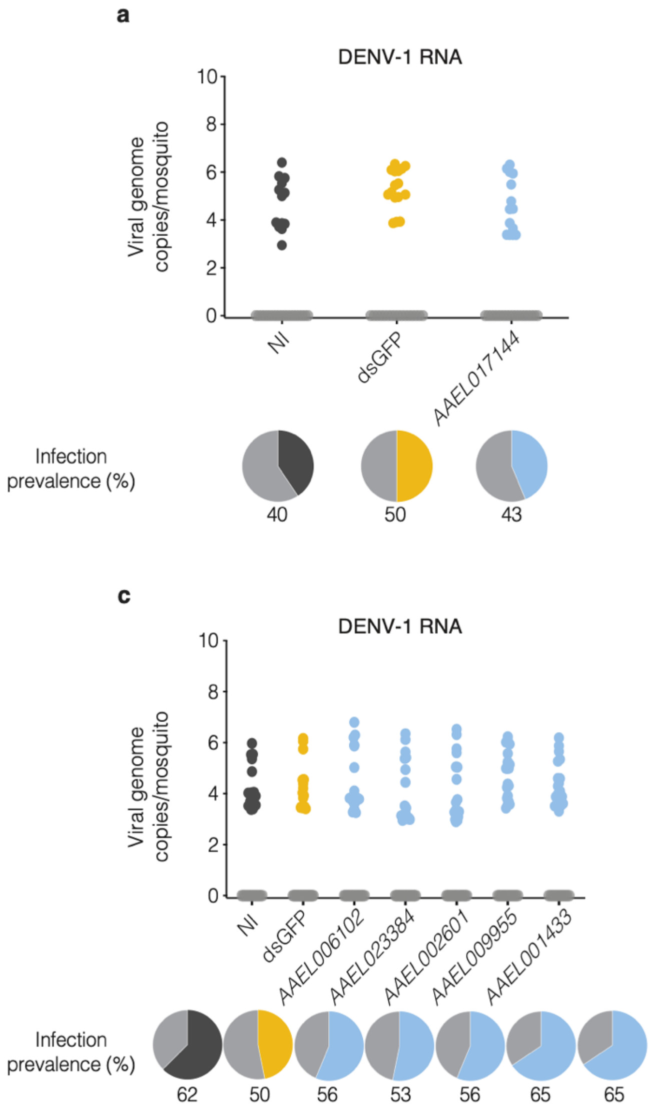

Figure S3. Shortlisted candidate genes not validated by gene silencing assays (related to Fig. 1). DENV-1 RNA levels and infection prevalence in whole mosquitoes upon gene silencing of each candidate gene (by injection of dsRNA targeting each candidate) and DENV-1 exposure. Mosquitoes injected with dsRNA targeting *GFP* (dsGFP) or non-injected (NI) were included as controls. Viral RNA was quantified 5 days post DENV-1 exposure (7 days post dsRNA injection). The three panels (**a**-**c**) represent three independent experiments. In each panel, the graph shows viral RNA levels, and the pie charts below represent the proportion of positive individuals. Source data are provided as a Source Data file.

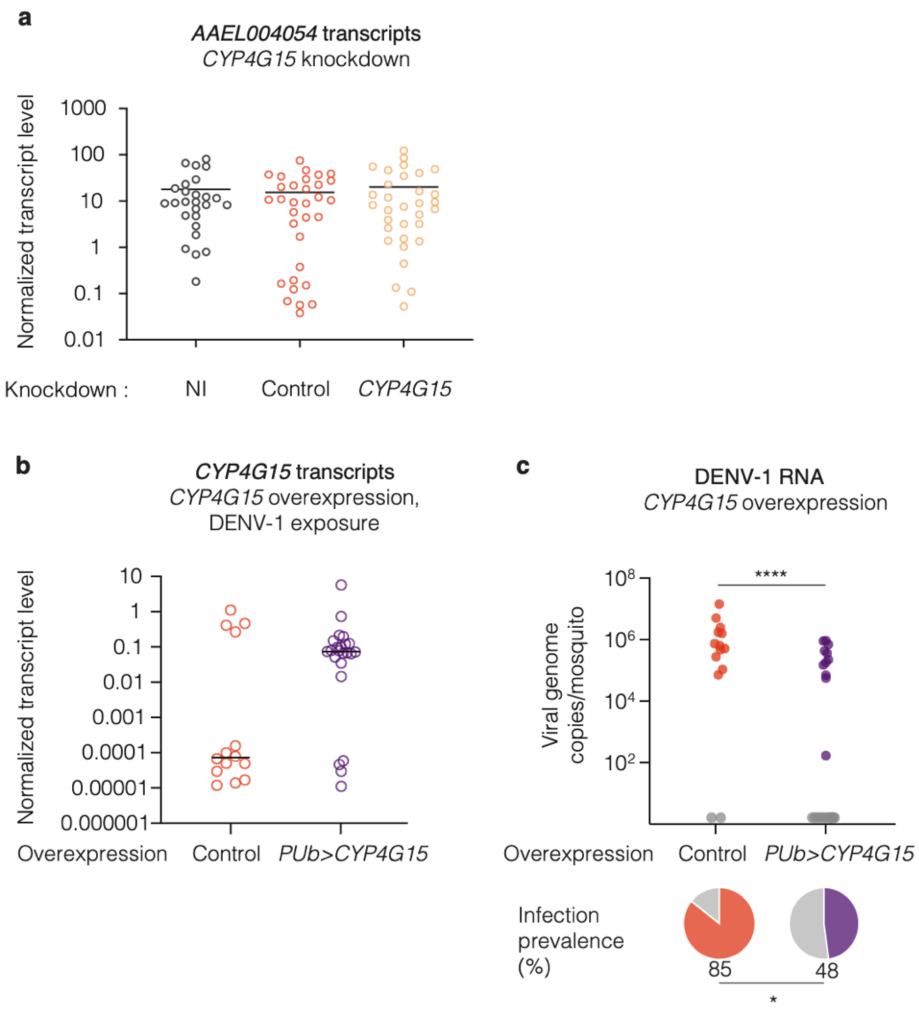

Figure S4. Controls of *CYP4G15* knockdown and overexpression experiments (related to Fig. 2). (**a**) *CYP4G36* (*AAEL004054*) expression in whole mosquitoes upon systemic gene silencing, 2 days after dsRNA injection targeting *CYP4G15* or *GFP*. Non-injected (NI) mosquitoes were also included. (**b**) *CYP4G15* expression in whole mosquitoes upon systemic *CYP4G15* overexpression and DENV-1 exposure. (**c**) DENV-1 RNA levels and infection prevalence in whole mosquitoes upon systemic *CYP4G15* overexpression and DENV-1 exposure. The graph shows viral RNA levels, and the pie charts below represent the proportion of positive individuals. Statistical significance of the differences was assessed with Mann-Whitney's test for the viral RNA levels and a chi-squared test for infection prevalence (*p<0.05; ****p<0.0001). In (**a-c**), the control line was the corresponding wild-type mosquito strain. In (**b-c**), *CYP4G15* was overexpressed transgenically under the control of a *Polyubiquitin* (*PUb*) promoter and mosquitoes were tested 5 days after a bloodmeal containing 2.5×10^7 FFU/ml of DENV-1. In (**a-b**), relative expression was calculated as $2^{-\Delta Ct}$, where $\Delta Ct = Ct_{Gene} - Ct_{RP49}$, using the housekeeping gene *RP49* for normalization. Statistical significance of the differences was assessed with Mann-Whitney's test, and the horizontal bars represent the medians. Source data are provided as a Source Data file.

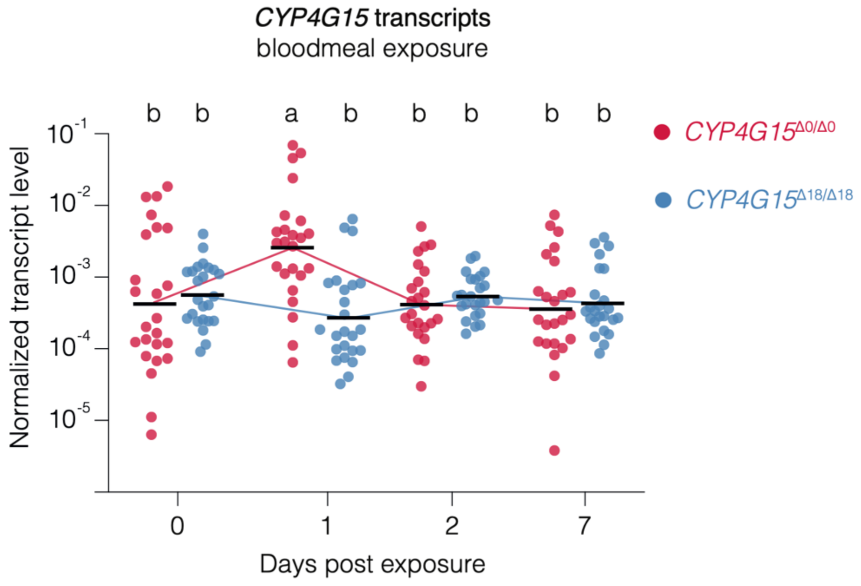

Figure S5. Kinetics of *CYP4G15* expression following a bloodmeal. The graph shows the time course of *CYP4G15* expression in the midguts of mosquitoes from sub-strains homozygous for the $CYP4G15^{\Delta 18}$ and $CYP4G15^{\Delta 0}$ variants following a non-infectious bloodmeal. Mosquitoes on day 0 are starved and sampled prior to the bloodmeal. Relative gene expression was calculated as $2^{-\Delta Ct}$, where $\Delta Ct = Ct_{CYP4G15} - Ct_{RPS17}$, using the housekeeping gene *RPS17* for normalization. Groups of 24 mosquitoes per condition were analyzed and statistical significance of the pairwise differences was determined by one-way ANOVA after $\log_{10}$-transformation of the $2^{-\Delta Ct}$ values, followed by Tukey-Kramer's HSD test. Statistical significance is represented above the graph using letters; groups that do not share a letter are significantly different ($p<0.05$). The horizontal bars represent the means. Source data are provided as a Source Data file.

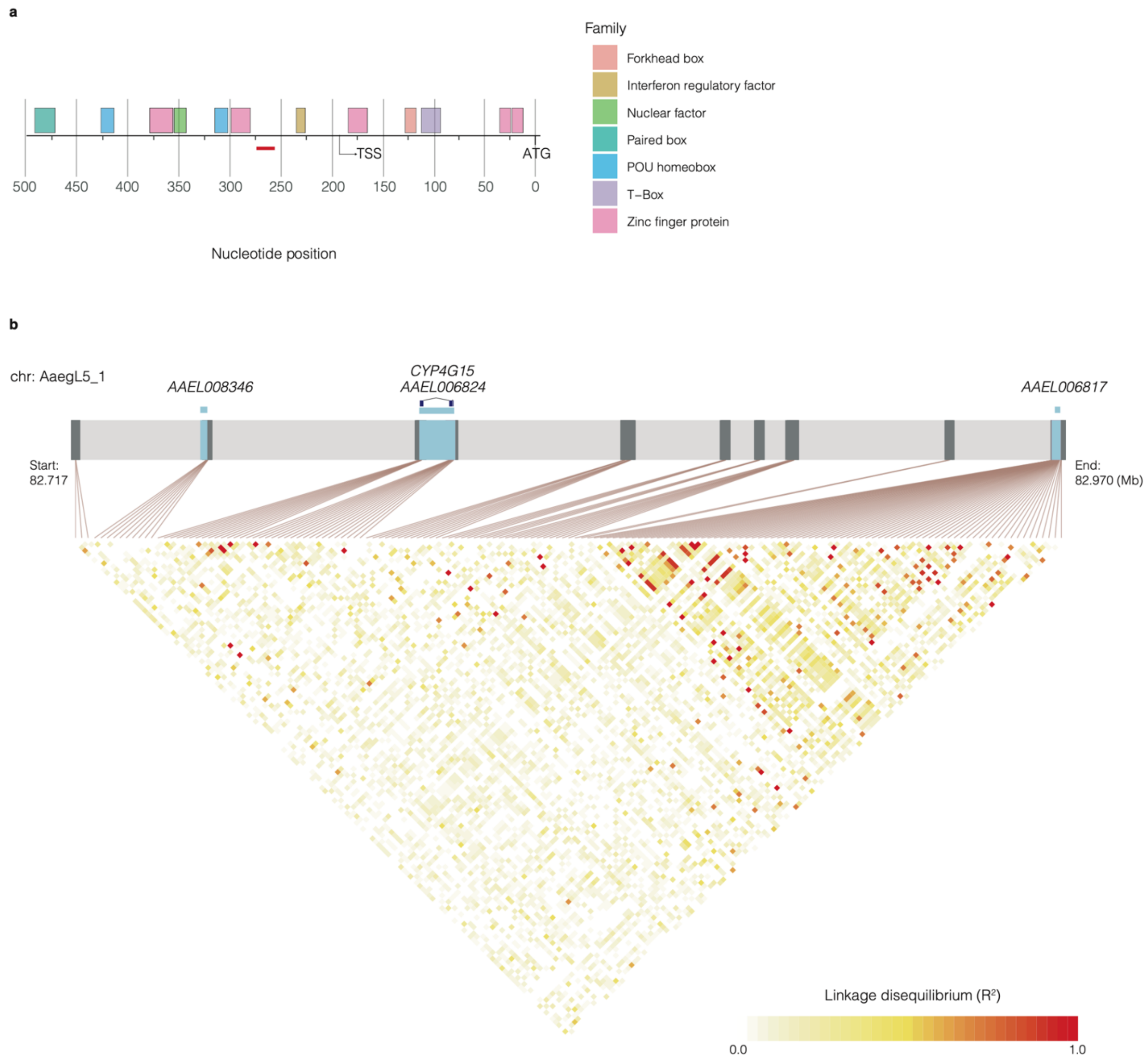

Figure S6. Genomic analysis of the *CYP4G15* locus. (**a**) Transcription factor binding motifs identified 500 bp upstream of the *CYP4G15* coding region. Nucleotide positions are numbered relative to the start codon (ATG) and the transcription start site (TSS) is indicated. Motif hits are classified by transcription factor family. The horizontal red bar represents the position of the Δ18 deletion. (**b**) Linkage disequilibrium (LD) patterns in the genomic region surrounding the *CYP4G15* gene were analyzed using whole-genome sequencing data available for 43 wild *Ae. aegypti* specimens from Gabon. In the horizontal bar at the top, light grey boxes represent repeat regions (excluded from the LD analysis) whereas blue boxes represent genes, and dark grey boxes represent non-repeat, non-gene regions. The matrix below shows LD (R^2) for each pair of SNPs, connected to their genomic positions with brown lines.

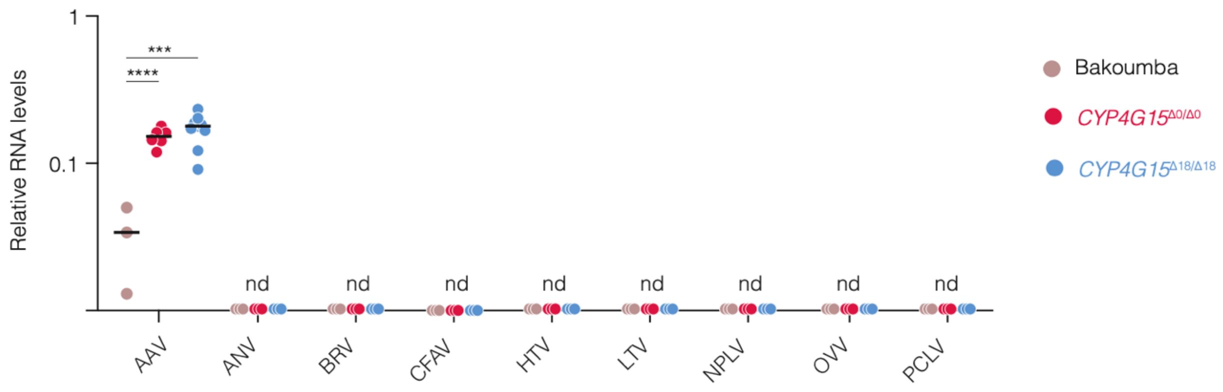

Figure S7. Detection of insect-specific viruses. A panel of 9 common insect-specific viruses in *Ae. aegypti* (AAV: *Aedes* anphevirus; ANV: Aslam narnavirus; BRV: Bahianus rhabdovirus; CFAV: cell-fusing agent virus; HTV: Humaita-Tubiacanga virus; LTV: Lactea totivirus; NPLV: Nyamuk partiti-like virus; OVV: Orbis virgavirus; PCLV: Phasi Charoen-like phasivirus) were detected by RT-qPCR in pools of 15 mosquitoes from the Bakoumba strain and the two sub-strains homozygous for the *CYP4G15*$^{\Delta 18}$ and *CYP4G15*$^{\Delta 0}$ variants. Relative viral RNA levels were calculated as $2^{-\Delta Ct}$, where $\Delta Ct = Ct_{Virus} - Ct_{RPS17}$, using the housekeeping gene *RPS17* for normalization. Statistical significance of the differences was assessed with Mann-Whitney's test (***p<0.001; ****p<0.0001). The horizontal bars represent the medians. nd = not detected. Source data are provided as a Source Data file.

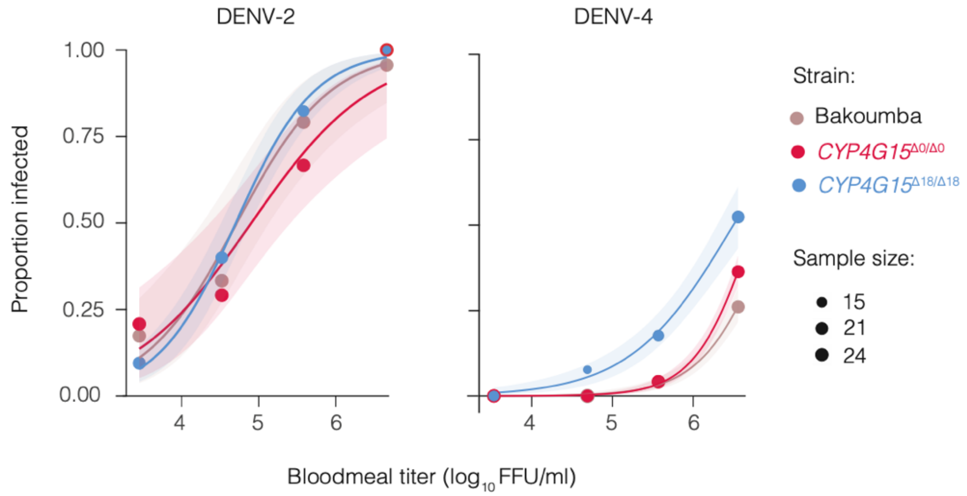

Figure S8. Susceptibility of *CYP4G15* sub-strains to DENV-2 and DENV-4 infection. Dose-response curves for DENV-2 (left) and DENV-4 (right) infection of the Bakoumba strain and two sub-strains homozygous for the *CYP4G15*$^{\Delta 18}$ and *CYP4G15*$^{\Delta 0}$ variants, respectively. The proportion of mosquitoes positive for viral RNA 7 days post DENV exposure are shown as a function of the bloodmeal titer in $\log_{10}$-transformed focus-forming units (FFU)/ml. The size of the symbols is proportional to the sample size (n=15-24 mosquitoes each). Curves are logistic regressions of the data with their 95% confidence intervals indicated by shaded bands. The full statistical analysis of the dose-response curves is provided in Table S1. Source data are provided as a Source Data file.

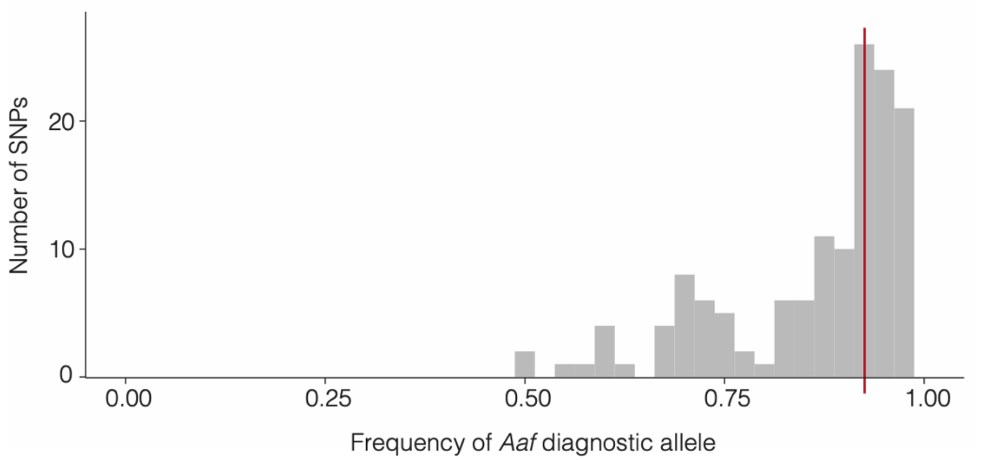

Figure S9. Subspecies assignment of the Bakoumba strain. The figure shows the allele frequency spectrum of the Bakoumba strain for 154 diagnostic SNPs identified as highly discriminant between *Ae. aegypti formosus* (*Aaf*) and *Ae. aegypti aegypti* (*Aaa*). The vertical red line indicates the median frequency of *Aaf* diagnostic alleles in the Bakoumba strain (0.925). Source data are provided as a Source Data file.

Table S1. Statistical tests of the dose-response curves shown in Fig. 4b and Fig. S8. For each virus, infection prevalence was analyzed by logistic regression as a function of replicate experiment, mosquito sub-strain, infectious dose, and all their interactions. Statistical significance of the effects was evaluated using a likelihood ratio (LR) test. The table shows the minimal adequate model after sequentially removing non-significant terms ($p<0.05$). Infectious dose (bloodmeal titer) was $\log_{10}$-transformed prior to the analysis. Df = degrees of freedom.

	DENV-1			DENV-3			DENV-2			DENV-4		
**Variable**	**Df**	**LR χ^2**	***P* value**	**Df**	**LR χ^2**	***P* value**	**Df**	**LR χ^2**	***P* value**	**Df**	**LR χ^2**	***P* value**
Experiment	2	13.50	0.0012	2	65.07	<0.0001						
Strain	2	20.84	<0.0001	2	40.73	<0.0001				2	7.009	0.0301
Experiment*Strain	4	9.651	0.0467									
Dose	1	194.2	<0.0001	1	240.5	<0.0001	1	127.5	<0.0001	1	53.31	<0.0001
Strain*Dose	2	16.79	0.0002									

Table S2. List of oligonucleotides used in this study

Application	Name	Sequence (5'-3')
**Transgenesis**[a]		
CYP4G15 promoter amplification	P1	TTCGATATCggtctcg**atcC**GGTATATCTTGCGGTTGTT
	P2	GATAAGCTTggtctcg**cata**TTGTAAATCACWCCGCTCAC
Polyubiquitin promoter amplification	P3	GGTCTCAcattGTTGAAATCTCTGTTGAGCAGAAAAAGAAACGA
	P4	GGTCTCgaATGGCCTCGCCCCTGAC
CYP4G15 open reading frame amplification	P5	ggtctcgAATGAGCGCGGAAATTGTGGCCGA
	P6	ggtctcaGACTCTCGGTTCCAGCTGTATTCTGA
CYP4G15 terminator amplification	P7	ggtctcg**AGTC**TAATCAATTATGACCACGAACTGAG
	P8	ggtctca**cccc**AATCCCAGGAAGCATGTTTTGG
**DENV RNA quantification**		
DENV-3 TaqMan probe	P9	FAM-ACATCTCTAAGATACCCGGAGGAG-BHQ1
DENV-1 TaqMan probe	P10	FAM-CTCAGAGACATATCAAAGATTCCAGGG-BHQ1
DENV-3 qPCR primers	P11	AGAAGGAGAAGGACTGCACA
	P12	ATTCTTGTGTCCCAACCGGCT
DENV-1 qPCR primers	P13	GGAAGGAGAAGGACTCCACA
	P14	ATCCTTGTATCCCATCCGGCT
**DENV RNA qualitative detection**		
DENV-3 PCR primers	P15	AGAAGGAGAAGGACTGCACA
	P16	ACCTGTCCACTGCCTCTTTG
DENV-1 PCR primers	P17	CGAAGATCACTGGTTCAGCA
	P18	ACATCCATCACGGTTCCATT
**Gene expression measurement**		
RP49 (*AAEL003396*) qPCR primers	P19	ACAAGCTTGCCCCCAACT
	P20	CCGTAACCGATGTTTGGC
RPS17 (*AAEL004175*) qPCR primers	P21	AAGAAGTGGCCATCATTCCA
	P22	GGTCTCCGGGTCGACTTC
CYP4G15 (*AAEL006824*) regular qPCR primers	P23	AGGGATTCGACTATGCGATG
	P24	GGTGAAGTTGAAGACGGAGTC
CYP4G15 (*AAEL006824*) degenerate qPCR primers	P25	AGGGATTYGAYTATGCGATG
	P26	GGTGAAGTTGAAGACRGAGTC
GFP qPCR primers	P27	ACGTAAACGGCCACAAGTTC
	P28	TGAACTTCAGGGTCAGCTTG
CYP4G15 (*AAEL004054*) qPCR primers	P29	AACACCAATAGCGTGGAAGG
	P30	ACCGTTTTCTGCGCTTTCTA
**Double-stranded RNA synthesis**[b]		
T7-*GFP* primers	P31	gaattgtaatacgactcactatagggCATGGTGAGCAAGGGCGAG
	P32	gaattgtaatacgactcactatagggCTTACTTGTACAGCTCGTC
TAG-*CYP4G15* primers for 1st PCR step	P33	GCCCGACGCggtcgtcaagcagaagaagg
	P34	CGCCTCGGCgacggacatcttgggtcact
T7-TAG primers for 2nd PCR step	P35	taatacgactcactatagggGCCCGACGC
	P36	taatacgactcactatagggCGCCTCGGC
**Insect-specific virus RNA quantification**		
Aedes anphevirus qPCR primers	P37	ATGAGGGCTGCTTTTCTTCG
	P38	ATTCGTGCAGCTTGCTTGAG
Aslam narnavirus qPCR primers[c]	P39	CTCAGGATTGAAGACGCGGT
	P40	GGCCCAGATTGTAGGATCCG
Bahianus rhabdovirus qPCR primers[c]	P41	TGAGCATCATCGGATCCACG
	P42	GCGCTTCCCTCCAAGTAACT
Cell-fusing agent virus qPCR primers[c]	P43	ACACGAGTGAAGCTGGTTGA
	P44	ACATACGTTCCTGGTTCCCG

Humaita-Tubiacanga virus qPCR primers[c]	P45	TGGCGATGCACATTCTAGCT
	P46	AACTTCCTGCACGACGTCAT
Lactea totivirus qPCR primers[c]	P47	ATGGTCGTGATGTGTATGAG
	P48	TCTAGCGGGATATGCTGATA
Nyamuk partiti-like virus qPCR primers[c]	P49	ACAGTATCGGCGCAGTTGAT
	P50	CACCAGGTAGTCCCATGCTC
Orbis virgavirus qPCR primers[c]	P51	CTCCACGACTTTGACCGACA
	P52	AACGGAGCAGTCATCCACAG
Phasi Charoen-like phasivirus qPCR primers[c]	P53	AGACAAGAAGAGTTGAGTGTGCT
	P54	AGCAACGTAATTAGTCCCTCCA
***CYP4G15* promoter genotyping**		
CYP4G15 promoter amplification	P55	ACGACAAAATGCTTCCCACT
	P56	GCCGTCTCCAGCAGAATATCC
CYP4G15 promoter sequencing	P57	CTCGCTGGACTTTGTCAGGT

[a]*Bsa*I recognition sequences are underlined, overhang sequences are in bold.
[b]T7 sequences are in small caps.
[c]Primer sequences obtained from Olmo, R. P. *et al.* (2023) *Nat Microbiol* **8**, 135-149.
