# Supplementary material for: Dengue virus susceptibility in Aedes aegypti linked to natural cytochrome P450 promoter variants
Source: Nat Commun. 2025 Aug 12;16:7468. doi: 10.1038/s41467-025-62693-y (PMC12343897; doi:10.1038/s41467-025-62693-y)
Supplement: Supplementary file 2 — Description of Additional Supplementary Files [file 41467_2025_62693_MOESM2_ESM.pdf]

Description of Additional Supplementary Files

File Name: Supplementary Data 1
Description: List of significantly differentially expressed genes between DENV-1 (D1)-infected and uninfected midguts (fold change >2, adjusted p value <0.05) in four separate tabs. For each time point (24 and 48 hours post exposure), the identifiers of genes significantly upregulated (UP) and downregulated (DOWN) in infected (H) relative to uninfected (L) mosquitoes are provided together with their raw and normalized read counts, fold change, raw p value, and adjusted p value using Benjamini and Hochberg's correction for multiple testing. Experimental conditions are labelled as D1- 24-H, D1-24-L, D1-48-H, D1-48-L and include 8 replicates numbered 1-8 for each condition. Column A shows the genes selected for functional testing, column B shows gene identifiers inherent to the study, including genes encoding novel transcripts labeled as TRXXXX. Column C shows NCBI gene identifiers labeled as LOCXXXXXX, and non-annotated genes are referred to as NA. Column D shows VectorBase gene identifiers labelled as AAELXXXXXX, and non-annotated genes are referred to as NA. Columns E-G show genomic coordinates, column H shows genomic strand orientation. Columns I-L show available gene descriptions from NCBI and VectorBase, and column H shows predicted annotations of new transcripts generated in this study.

File Name: Supplementary Data 2
Description: Plasmid sequences used for the construction of the *Prom*$^{\Delta 0}$ *>GFP* reporter, *Prom*$^{\Delta 18}$*>GFP* reporter, and PUb>CYP4G15 overexpression transgenes.
