# Supplementary material for: Dengue virus susceptibility in Aedes aegypti linked to natural cytochrome P450 promoter variants
Source: Nat Commun. 2025 Aug 12;16:7468. doi: 10.1038/s41467-025-62693-y (PMC12343897; doi:10.1038/s41467-025-62693-y)
Supplement: Supplementary file 4 — Supplementary Data 2 [file 41467_2025_62693_MOESM4_ESM.docx]

1. ***Prom*^Δ0^>*GFP* reporter plasmid**

LOCUS #1370_GGC_HAP1-G 5899 bp DNA circular SYN 30-JAN-2024

DEFINITION synthetic circular DNA

ACCESSION .

VERSION .

KEYWORDS .

SOURCE synthetic DNA construct

ORGANISM recombinant plasmid

REFERENCE 1 (bases 1 to 5899)

AUTHORS

TITLE Direct Submission

JOURNAL Exported Jan 30, 2024 from SnapGene 6.2.2

https://www.snapgene.com

FEATURES Location/Qualifiers

source 1..5899

/mol_type="other DNA"

/organism="recombinant plasmid"

misc_feature 271..286

/label=M13F

/note="M13F"

primer_bind 467..490

/label=EM2063

primer_bind 679..699

/label=EM2473

primer_bind complement(1115..1135)

/label=EM2474

primer_bind complement(1205..1232)

/label=EM2064

CDS 1228..1941

/codon_start=1

/product="enhanced GFP"

/label=EGFP

/note="mammalian codon-optimized"

/label=SV40 poly(A) signal

/note="SV40 polyadenylation signal"

misc_feature 2191..2417

/label=SV40 term

/note="SV40 term"

misc_feature 2418..2694

/label=attB

/note="attB"

misc_feature 2768..2817

/label=3x Pax6 binding sites

/note="3x Pax6 binding sites"

misc_feature 2857..2864

/label=TATA

/note="TATA"

misc_feature 3027..3708

/label=DsRed

/note="DsRed"

misc_feature 3710..3950

/label=SV40 term

/note="SV40 term"

/note="attL2"

misc_feature complement(4160..4178)

/label=M13R

/note="M13R"

ORIGIN

1 ctttcctgcg ttatcccctg attctgtgga taaccgtatt accgcctttg agtgagctga

61 taccgctcgc cgcagccgaa cgaccgagcg cagcgagtca gtgagcgagg aagcggaaga

121 gcgcccaata cgcaaaccgc ctctccccgc gcgttggccg attcattaat gcagctggca

181 cgacaggttt cccgactgga aagcgggcag tgagcgcaac gcaattaata cgcgtaccgc

241 tagcatggat gttttcccag tcacgacgtt gtaaaacgac ggccagtctt aagctcgggc

301 ccctacaggt cactaatacc atctaagtag ttgattcata gtgactggat atgttgtgtt

361 ttacagtatt atgtagtctg ttttttatgc aaaatctaat ttaatatatt gatatttata

421 tcattttacg tttctcgttc aacttttcta tacaaagttg gtaccggatc cggtatatct

481 tgcggttgtt attcgacttt gctttagtac tgtaccggta atcgaaacta accactttta

541 gaatggcctt gctttacaat tgcgtagtta ccaactaagc tcaacaaagg ccattcaata

601 acctttttta cagaatcagc atcagaatca tggcaatgat aaaaaaatgt tgaagcattt

661 caaacttcgc caataactca tccattccga gcactatgtg caaatgagat cgctttcagt

721 acctcaaccc tgggttactc ccggctgccc atcgtcccgg gactacgtgg ctgtcggcag

781 ctactaaata cctacactaa ttataatgca ttcaaaagtt tatgggttcg ataaatttcg

841 atacgacgac aaaatgcttc ccactggaag cgctgaatca cctcccgtcc agccccgtcg

901 tcccgtccct ttgcatttgc aagtgcattt ccctcgctga actgcagcac cggtcggtgc

961 cggattgacg agcgaatttt taattaccac ttcgatttcg ccgcttataa atacaccgtc

1021 ccgggtcgta aattatccaa gtcgcattcg gaggtacagc agagcgtgcg cgtattccca

1081 gtgtgacgcg agaaacgacc aatcaaacga cggaagcttg agagtgtgaa attcggtttt

1141 cggtggggcc gtcaggctta gaaaaggggc ttagaagaag gatctcgatc cgcgtttttc

1201 cggtgtgagc ggwgtgattt acaatatgtg agcaagggcg aggagctgtt caccggggtg

1261 gtgcccatcc tggtcgagct ggacggcgac gtaaacggcc acaagttcag cgtgtccggc

1321 gagggcgagg gcgatgccac ctacggcaag ctgaccctga agttcatctg caccaccggc

1381 aagctgcccg tgccctggcc caccctcgtg accaccctga cctacggcgt gcagtgcttc

1441 agccgctacc ccgaccacat gaagcagcac gacttcttca agtccgccat gcccgaaggc

1501 tacgtccagg agcgcaccat cttcttcaag gacgacggca actacaagac ccgcgccgag

1561 gtgaagttcg agggcgacac cctggtgaac cgcatcgagc tgaagggcat cgacttcaag

1621 gaggacggca acatcctggg gcacaagctg gagtacaact acaacagcca caacgtctat

1681 atcatggccg acaagcagaa gaacggcatc aaggtgaact tcaagatccg ccacaacatc

1741 gaggacggca gcgtgcagct cgccgaccac taccagcaga acacccccat cggcgacggc

1801 cccgtgctgc tgcccgacaa ccactacctg agcacccagt ccgccctgag caaagacccc

1861 aacgagaagc gcgatcacat ggtcctgctg gagttcgtga ccgccgccgg gatcactctc

1921 ggcatggacg agctgtacaa gtaaagcggc cgcgactcta gatcaaatca gccataccac

1981 atttgtagag gttttacttg ctttaaaaaa cctcccacac ctccccctga acctgaaaca

2041 taaaatgaat gcaattgttg ttgttaactt gtttattgca gcttataatg gttacaaata

2101 aagcaatagc atcacaaatt tcacaaataa agcatttttc ttcactgcat tctagttgtg

2161 gtttgtccaa actcatcaat gtatcgcttc tagacataat cagccatacc acatttgtag

2221 aggttttact tgctttaaaa aacctcccac acctccccct gaacctgaaa cataaaatga

2281 atgcaattgt tgttgttaac ttgtttattg cagcttataa tggttacaaa taaagcaata

2341 gcatcacaaa tttcacaaat aaagcatttt tcttcactgc attctagttg tggtttgtcc

2401 aaactcatca atgtatctcg acgatgtagg tcacagtctc gaagccgcgg tgcgggtgcc

2461 agggcgtgcc cttgggctcc ccgggcgcgt actccacctc acccatctgg tccatcatga

2521 tgaacgggtc gaggtggcgg tagttgatcc cggcgaacgc gcggcgcacc gggaagccct

2581 cgccctcgaa accgctgggc gcggtggtca cggtgagcac gggacgtgcg acggcgtcgg

2641 cgggtgcgga tacgcggggc agcgtcagcg ggttctcgac ggtcacggcg ggcaattcct

2701 gcagacttcc ggtatctcgc gtttgtttga tcgcacggtt cccacaatgg ttaattcgag

2761 ctcgcccggg gatctaattc aattagagac taattcaatt agagctaatt caattaggat

2821 ccaagcttat cgatttcgaa ccctcgaccg ccggagtata aatagaggcg cttcgtctac

2881 ggagcgacaa ttcaattcaa acaagcaaag tgaacacgtc gctaagcgaa agctaagcaa

2941 ataaacaagc gcagctgaac aagctaaaca atcggggtac cgctagagtc gacggtaccg

3001 cgggcccggg atccaccggt cgccaccatg gtgcgctcct ccaagaacgt catcaaggag

3061 ttcatgcgct tcaaggtgcg catggagggc accgtgaacg gccacgagtt cgagatcgag

3121 ggcgagggcg agggccgccc ctacgagggc cacaacaccg tgaagctgaa ggtgaccaag

3181 ggcggccccc tgcccttcgc ctgggacatc ctgtcccccc agttccagta cggctccaag

3241 gtgtacgtga agcaccccgc cgacatcccc gactacaaga agctgtcctt ccccgagggc

3301 ttcaagtggg agcgcgtgat gaacttcgag gacggcggcg tggtgaccgt gacccaggac

3361 tcctccctgc aggacggctg cttcatctac aaggtgaagt tcatcggcgt gaacttcccc

3421 tccgacggcc ccgtaatgca gaagaagacc atgggctggg aggcctccac cgagcgcctg

3481 tacccccgcg acggcgtgct gaagggcgag atccacaagg ccctgaagct gaaggacggc

3541 ggccactacc tggtggagtt caagtccatc tacatggcca agaagcccgt gcagctgccc

3601 ggctactact acgtggactc caagctggac atcacctccc acaacgagga ctacaccatc

3661 gtggagcagt acgagcgcac cgagggccgc caccacctgt tcctgtagcg gccgcgactc

3721 tagatcataa tcagccatac cacatttgta gaggttttac ttgctttaaa aaacctccca

3781 cacctccccc tgaacctgaa acataaaatg aatgcaattg ttgttgttaa cttgtttatt

3841 gcagcttata atggttacaa ataaagcaat agcatcacaa atttcacaaa taaagcattt

3901 ttttcactgc attctagttg tggtttgtcc aaactcatca atgtatctta aagcttatcg

3961 atacgcgtac ggcgcgccta gagcggccgc caccgcggtg gagctcgagt acccagcttt

4021 cttgtacaaa gttggcatta taagaaagca ttgcttatca atttgttgca acgaacaggt

4081 cactatcagt caaaataaaa tcattatttg ccatccagct gcagggcggc cgcgatatcc

4141 cctatagtga gtcgtattac atggtcatag ctgtttcctg gcagctctgg cccgtgtctc

4201 aaaatctctg atgttacatt gcacaagata aaaatatatc atcatgaaca ataaaactgt

4261 ctgcttacat aaacagtaat acaaggggtg ttatgagcca tattcaacgg gaaacgtcga

4321 ggccgcgatt aaattccaac atggatgctg atttatatgg gtataaatgg gctcgcgata

4381 atgtcgggca atcaggtgcg acaatctatc gcttgtatgg gaagcccgat gcgccagagt

4441 tgtttctgaa acatggcaaa ggtagcgttg ccaatgatgt tacagatgag atggtcagac

4501 taaactggct gacggaattt atgcctcttc cgaccatcaa gcattttatc cgtactcctg

4561 atgatgcatg gttactcacc actgcgatcc ccggaaaaac agcattccag gtattagaag

4621 aatatcctga ttcaggtgaa aatattgttg atgcgctggc agtgttcctg cgccggttgc

4681 attcgattcc tgtttgtaat tgtcctttta acagcgatcg cgtatttcgt ctcgctcagg

4741 cgcaatcacg aatgaataac ggtttggttg atgcgagtga ttttgatgac gagcgtaatg

4801 gctggcctgt tgaacaagtc tggaaagaaa tgcataaact tttgccattc tcaccggatt

4861 cagtcgtcac tcatggtgat ttctcacttg ataaccttat ttttgacgag gggaaattaa

4921 taggttgtat tgatgttgga cgagtcggaa tcgcagaccg ataccaggat cttgccatcc

4981 tatggaactg cctcggtgag ttttctcctt cattacagaa acggcttttt caaaaatatg

5041 gtattgataa tcctgatatg aataaattgc agtttcattt gatgctcgat gagtttttct

5101 aatcagaatt ggttaattgg ttgtaacact ggcagagcat tacgctgact tgacgggacg

5161 gcgcaagctc atgaccaaaa tcccttaacg tgagttacgc gtcgttccac tgagcgtcag

5221 accccgtaga aaagatcaaa ggatcttctt gagatccttt ttttctgcgc gtaatctgct

5281 gcttgcaaac aaaaaaacca ccgctaccag cggtggtttg tttgccggat caagagctac

5341 caactctttt tccgaaggta actggcttca gcagagcgca gataccaaat actgttcttc

5401 tagtgtagcc gtagttaggc caccacttca agaactctgt agcaccgcct acatacctcg

5461 ctctgctaat cctgttacca gtggctgctg ccagtggcga taagtcgtgt cttaccgggt

5521 tggactcaag acgatagtta ccggataagg cgcagcggtc gggctgaacg gggggttcgt

5581 gcacacagcc cagcttggag cgaacgacct acaccgaact gagataccta cagcgtgagc

5641 tatgagaaag cgccacgctt cccgaaggga gaaaggcgga caggtatccg gtaagcggca

5701 gggtcggaac aggagagcgc acgagggagc ttccaggggg aaacgcctgg tatctttata

5761 gtcctgtcgg gtttcgccac ctctgacttg agcgtcgatt tttgtgatgc tcgtcagggg

5821 ggcggagcct atggaaaaac gccagcaacg cggccttttt acggttcctg gccttttgct

5881 ggccttttgc tcacatgtt

//

1. ***Prom*^Δ18^>*GFP* reporter plasmid**

LOCUS #1369_GGC_HAP3-G 5882 bp DNA circular SYN 30-JAN-2024

DEFINITION synthetic circular DNA

ACCESSION .

VERSION .

KEYWORDS .

SOURCE synthetic DNA construct

ORGANISM synthetic DNA construct

REFERENCE 1 (bases 1 to 5882)

AUTHORS

TITLE Direct Submission

JOURNAL Exported Jan 30, 2024 from SnapGene 6.2.2

https://www.snapgene.com

FEATURES Location/Qualifiers

source 1..5882

/mol_type="other DNA"

/organism="synthetic DNA construct"

misc_feature 1..467

/label=seq478043

/note="seq478043"

primer_bind 271..287

/label=M13 fwd

/note="common sequencing primer, one of multiple similar

variants"

misc_feature 271..286

/label=M13F

/note="M13F"

misc_feature 465..467

/label=EM156

/note="EM156"

primer_bind 679..699

/label=EM2473

primer_bind complement(1097..1117)

/label=EM2474

primer_bind 1207..1224

/label=EM692

CDS 1208..1927

/codon_start=1

/product="the original enhanced GFP (Yang et al., 1996)"

/label=EGFP

/note="mammalian codon-optimized"

polyA_signal 2049..2131

/label=SV40 poly(A) signal

/note="SV40 polyadenylation signal"

primer_bind complement(2149..2178)

/label=EM634

misc_feature 2174..2400

/label=SV40 term

/note="SV40 term"

polyA_signal 2281..2363

/label=SV40 poly(A) signal

/note="SV40 polyadenylation signal"

misc_feature 2401..2677

/label=attB

/note="attB"

protein_bind 2421..2490

/label=attB

/bound_moiety="phage phi-C31 integrase"

/note="attB site for the phi-C31 integrase (Groth et al.,

2000)"

misc_feature 2751..2800

/label=3x Pax6 binding sites

/note="3x Pax6 binding sites"

misc_feature 2840..2847

/label=TATA

/note="TATA"

regulatory 3005..3014

/label=Kozak sequence

/note="vertebrate consensus sequence for strong initiation

of translation (Kozak, 1987)"

/regulatory_class="other"

misc_feature 3010..3691

/label=DsRed

/note="DsRed"

CDS 3011..3691

/codon_start=1

/product="wild-type DsRed"

/label=DsRed1

/note="mammalian codon-optimized"

misc_feature 3693..3933

/label=SV40 term

/note="SV40 term"

polyA_signal 3812..3933

/label=SV40 poly(A) signal

/note="SV40 polyadenylation signal"

promoter complement(4124..4142)

/label=T7 promoter

/note="promoter for bacteriophage T7 RNA polymerase"

misc_feature complement(4143..4161)

/label=M13R

/note="M13R"

primer_bind complement(4147..4163)

/label=M13 rev

CDS 4276..5085

/codon_start=1

/gene="aph(3')-Ia"

/product="aminoglycoside phosphotransferase"

/label=KanR

/note="confers resistance to kanamycin in bacteria or G418

(Geneticin(R)) in eukaryotes"

rep_origin 5232..5820

/direction=RIGHT

/label=ori

/note="high-copy-number ColE1/pMB1/pBR322/pUC origin of

replication"

misc_feature 5614..5882

/label=seq478043

/note="seq478043"

ORIGIN

1 ctttcctgcg ttatcccctg attctgtgga taaccgtatt accgcctttg agtgagctga

61 taccgctcgc cgcagccgaa cgaccgagcg cagcgagtca gtgagcgagg aagcggaaga

121 gcgcccaata cgcaaaccgc ctctccccgc gcgttggccg attcattaat gcagctggca

181 cgacaggttt cccgactgga aagcgggcag tgagcgcaac gcaattaata cgcgtaccgc

241 tagcatggat gttttcccag tcacgacgtt gtaaaacgac ggccagtctt aagctcgggc

301 ccctacaggt cactaatacc atctaagtag ttgattcata gtgactggat atgttgtgtt

361 ttacagtatt atgtagtctg ttttttatgc aaaatctaat ttaatatatt gatatttata

421 tcattttacg tttctcgttc aacttttcta tacaaagttg gtaccggatc cggtatatct

481 tgcggttgtt attcgacttt gctttagtac tgtaccggta atcgaaacta accactttta

541 gaatggcctt gctttacaat tgcgtagtta ccaactaagc tcaacaaagg ccattcaata

601 acctttttta cagaatcagc atcagaatca tggcaatgat aaaaaaatgt tgaagcattt

661 caaacttcgc caataactca tccattccga gcactatgtg caaatgagat cgctttcagt

721 acctcaaccc tgggttactc ccggctgccc atcgtcccgg gactacgtgg ctgtcggcag

781 ctactaaata cctacactaa ttataatgca ttcaaaagtt tatgggttcg ataaatttcg

841 atacgacgac aaaatgcttc ccactggaag cgctgaatca cctcccgtcc agccccgtcg

901 tcccgtccct ttgcatttgc aagtgcattt ccctcgctga actgcagcac cgagcgaatt

961 tttaattacc acttcgattt cgccgcttat aaatacaccg tcccgggtcg taaattatcc

1021 aagtcgcatt cggaggtaca gcagagcgtg cgcgtattcc ctgtgtgacg cgagaaacga

1081 ccaatcaaac gacggaagct tgagagtgtg aaattcggtt ttcggtgggg ccgtcaggct

1141 tagaataggg gcttagaaga aggatctcga tccgcgtttt tccggtgtga gcggagtgat

1201 ttacaaaatg gtgagcaagg gcgaggagct gttcaccggg gtggtgccca tcctggtcga

1261 gctggacggc gacgtaaacg gccacaagtt cagcgtgtcc ggcgagggcg agggcgatgc

1321 cacctacggc aagctgaccc tgaagttcat ctgcaccacc ggcaagctgc ccgtgccctg

1381 gcccaccctc gtgaccaccc tgacctacgg cgtgcagtgc ttcagccgct accccgacca

1441 catgaagcag cacgacttct tcaagtccgc catgcccgaa ggctacgtcc aggagcgcac

1501 catcttcttc aaggacgacg gcaactacaa gacccgcgcc gaggtgaagt tcgagggcga

1561 caccctggtg aaccgcatcg agctgaaggg catcgacttc aaggaggacg gcaacatcct

1621 ggggcacaag ctggagtaca actacaacag ccacaacgtc tatatcatgg ccgacaagca

1681 gaagaacggc atcaaggtga acttcaagat ccgccacaac atcgaggacg gcagcgtgca

1741 gctcgccgac cactaccagc agaacacccc catcggcgac ggccccgtgc tgctgcccga

1801 caaccactac ctgagcaccc agtccgccct gagcaaagac cccaacgaga agcgcgatca

1861 catggtcctg ctggagttcg tgaccgccgc cgggatcact ctcggcatgg acgagctgta

1921 caagtaaagc ggccgcgact ctagatcaaa tcagccatac cacatttgta gaggttttac

1981 ttgctttaaa aaacctccca cacctccccc tgaacctgaa acataaaatg aatgcaattg

2041 ttgttgttaa cttgtttatt gcagcttata atggttacaa ataaagcaat agcatcacaa

2101 atttcacaaa taaagcattt ttcttcactg cattctagtt gtggtttgtc caaactcatc

2161 aatgtatcgc ttctagacat aatcagccat accacatttg tagaggtttt acttgcttta

2221 aaaaacctcc cacacctccc cctgaacctg aaacataaaa tgaatgcaat tgttgttgtt

2281 aacttgttta ttgcagctta taatggttac aaataaagca atagcatcac aaatttcaca

2341 aataaagcat ttttcttcac tgcattctag ttgtggtttg tccaaactca tcaatgtatc

2401 tcgacgatgt aggtcacagt ctcgaagccg cggtgcgggt gccagggcgt gcccttgggc

2461 tccccgggcg cgtactccac ctcacccatc tggtccatca tgatgaacgg gtcgaggtgg

2521 cggtagttga tcccggcgaa cgcgcggcgc accgggaagc cctcgccctc gaaaccgctg

2581 ggcgcggtgg tcacggtgag cacgggacgt gcgacggcgt cggcgggtgc ggatacgcgg

2641 ggcagcgtca gcgggttctc gacggtcacg gcgggcaatt cctgcagact tccggtatct

2701 cgcgtttgtt tgatcgcacg gttcccacaa tggttaattc gagctcgccc ggggatctaa

2761 ttcaattaga gactaattca attagagcta attcaattag gatccaagct tatcgatttc

2821 gaaccctcga ccgccggagt ataaatagag gcgcttcgtc tacggagcga caattcaatt

2881 caaacaagca aagtgaacac gtcgctaagc gaaagctaag caaataaaca agcgcagctg

2941 aacaagctaa acaatcgggg taccgctaga gtcgacggta ccgcgggccc gggatccacc

3001 ggtcgccacc atggtgcgct cctccaagaa cgtcatcaag gagttcatgc gcttcaaggt

3061 gcgcatggag ggcaccgtga acggccacga gttcgagatc gagggcgagg gcgagggccg

3121 cccctacgag ggccacaaca ccgtgaagct gaaggtgacc aagggcggcc ccctgccctt

3181 cgcctgggac atcctgtccc cccagttcca gtacggctcc aaggtgtacg tgaagcaccc

3241 cgccgacatc cccgactaca agaagctgtc cttccccgag ggcttcaagt gggagcgcgt

3301 gatgaacttc gaggacggcg gcgtggtgac cgtgacccag gactcctccc tgcaggacgg

3361 ctgcttcatc tacaaggtga agttcatcgg cgtgaacttc ccctccgacg gccccgtaat

3421 gcagaagaag accatgggct gggaggcctc caccgagcgc ctgtaccccc gcgacggcgt

3481 gctgaagggc gagatccaca aggccctgaa gctgaaggac ggcggccact acctggtgga

3541 gttcaagtcc atctacatgg ccaagaagcc cgtgcagctg cccggctact actacgtgga

3601 ctccaagctg gacatcacct cccacaacga ggactacacc atcgtggagc agtacgagcg

3661 caccgagggc cgccaccacc tgttcctgta gcggccgcga ctctagatca taatcagcca

3721 taccacattt gtagaggttt tacttgcttt aaaaaacctc ccacacctcc ccctgaacct

3781 gaaacataaa atgaatgcaa ttgttgttgt taacttgttt attgcagctt ataatggtta

3841 caaataaagc aatagcatca caaatttcac aaataaagca tttttttcac tgcattctag

3901 ttgtggtttg tccaaactca tcaatgtatc ttaaagctta tcgatacgcg tacggcgcgc

3961 ctagagcggc cgccaccgcg gtggagctcg agtacccagc tttcttgtac aaagttggca

4021 ttataagaaa gcattgctta tcaatttgtt gcaacgaaca ggtcactatc agtcaaaata

4081 aaatcattat ttgccatcca gctgcagggc ggccgcgata tcccctatag tgagtcgtat

4141 tacatggtca tagctgtttc ctggcagctc tggcccgtgt ctcaaaatct ctgatgttac

4201 attgcacaag ataaaaatat atcatcatga acaataaaac tgtctgctta cataaacagt

4261 aatacaaggg gtgttatgag ccatattcaa cgggaaacgt cgaggccgcg attaaattcc

4321 aacatggatg ctgatttata tgggtataaa tgggctcgcg ataatgtcgg gcaatcaggt

4381 gcgacaatct atcgcttgta tgggaagccc gatgcgccag agttgtttct gaaacatggc

4441 aaaggtagcg ttgccaatga tgttacagat gagatggtca gactaaactg gctgacggaa

4501 tttatgcctc ttccgaccat caagcatttt atccgtactc ctgatgatgc atggttactc

4561 accactgcga tccccggaaa aacagcattc caggtattag aagaatatcc tgattcaggt

4621 gaaaatattg ttgatgcgct ggcagtgttc ctgcgccggt tgcattcgat tcctgtttgt

4681 aattgtcctt ttaacagcga tcgcgtattt cgtctcgctc aggcgcaatc acgaatgaat

4741 aacggtttgg ttgatgcgag tgattttgat gacgagcgta atggctggcc tgttgaacaa

4801 gtctggaaag aaatgcataa acttttgcca ttctcaccgg attcagtcgt cactcatggt

4861 gatttctcac ttgataacct tatttttgac gaggggaaat taataggttg tattgatgtt

4921 ggacgagtcg gaatcgcaga ccgataccag gatcttgcca tcctatggaa ctgcctcggt

4981 gagttttctc cttcattaca gaaacggctt tttcaaaaat atggtattga taatcctgat

5041 atgaataaat tgcagtttca tttgatgctc gatgagtttt tctaatcaga attggttaat

5101 tggttgtaac actggcagag cattacgctg acttgacggg acggcgcaag ctcatgacca

5161 aaatccctta acgtgagtta cgcgtcgttc cactgagcgt cagaccccgt agaaaagatc

5221 aaaggatctt cttgagatcc tttttttctg cgcgtaatct gctgcttgca aacaaaaaaa

5281 ccaccgctac cagcggtggt ttgtttgccg gatcaagagc taccaactct ttttccgaag

5341 gtaactggct tcagcagagc gcagatacca aatactgttc ttctagtgta gccgtagtta

5401 ggccaccact tcaagaactc tgtagcaccg cctacatacc tcgctctgct aatcctgtta

5461 ccagtggctg ctgccagtgg cgataagtcg tgtcttaccg ggttggactc aagacgatag

5521 ttaccggata aggcgcagcg gtcgggctga acggggggtt cgtgcacaca gcccagcttg

5581 gagcgaacga cctacaccga actgagatac ctacagcgtg agctatgaga aagcgccacg

5641 cttcccgaag ggagaaaggc ggacaggtat ccggtaagcg gcagggtcgg aacaggagag

5701 cgcacgaggg agcttccagg gggaaacgcc tggtatcttt atagtcctgt cgggtttcgc

5761 cacctctgac ttgagcgtcg atttttgtga tgctcgtcag gggggcggag cctatggaaa

5821 aacgccagca acgcggcctt tttacggttc ctggcctttt gctggccttt tgctcacatg

5881 tt

//

1. ***PUb*>*CYP4G15* plasmid**

LOCUS Annas_PUB-P450 9226 bp DNA circular SYN 30-JAN-2024

DEFINITION synthetic circular DNA

ACCESSION .

VERSION .

KEYWORDS .

SOURCE synthetic DNA construct

ORGANISM synthetic DNA construct

REFERENCE 1 (bases 1 to 9226)

AUTHORS Eric Marois

TITLE Direct Submission

JOURNAL Exported Jan 30, 2024 from SnapGene 6.2.2

https://www.snapgene.com

FEATURES Location/Qualifiers

source 1..9226

/mol_type="other DNA"

/organism="synthetic DNA construct"

misc_feature 7..25

/label=M13R

/note="M13R"

misc_feature 62..197

/label=piggyBac 5' sequence

/note="piggyBac 5' sequence"

misc_feature complement(372..592)

/label=attP'

/note="attP'"

misc_feature complement(603..636)

/label=loxP

/note="loxP"

misc_feature 646..2026

/label=Aedes aegypti Polyubiquitin promoter

misc_feature 646..675

/label=PUb-fw

/note="PUb-fw"

3'UTR join(1234..1412,2017..2026)

intron 1413..2016

/note="3'UTRintron"

misc_feature complement(1994..2026)

/label=PUb rev

/note="PUb rev"

primer_bind 2027..2050

/label=EM1552

CDS 2028..3685

/codon_start=1

/label=P450 cds

/translation="MSAEIVAERGSSLVSLAVPMVIFMTLVLVASALFHFWMISRRYVQ

LGNKIPGPRAYPFIGNANMLLGMNHNEIMERAMQLSYIYGSVARGWLGYHLVVFLTEPA

DIEIILNSYVHLTKSSEYRFFKPWLGDGLLISSGEKWRSHRKLIAPAFHMNVLKTFVDV

FNDNSLAVVERMRKEVGKEFDVHDYMSEVTVDILLETAMGSQRTSESKEGFDYAMAVMK

MCDILHSRQLKFHLRMDSVFNFTKIKQEQERLLGIIHGLTRKVGKQKKELFEKNFADGK

LPSPSLSEIIAKEESESKEPLPVISQGSLLRDDLDFNDENDIGEKRRLAFLDLMIETAK

SGADLTDEEIKEEVDTIMFEGHDTTAAGSSFVLCLLGIHQDVQDRVYKEIYQIFGNSKR

KATFNDTLEMKYLERVIFETLRMYPPVPVIARKVTQDVRLASHDYVVPAGTTVVIGTYK

VHRRADIYPNPDVFNPDNFLPERTQNRHYYSYIPFSAGPRSCVGRKYAMLKLKVLLSTI

LRNYRVVSNLKESDFKLQGDIILKRTDGFRIQLEP"

primer_bind complement(3664..3685)

/label=EM1553

terminator 3690..4259

/label=P450 term

primer_bind 4260..4293

/label=EM1169

primer_bind 4260..4271

/label=EM 1998

misc_feature 4264..4293

/label=PUb-fw

/note="PUb-fw"

3'UTR join(4852..5030,5635..5644)

intron 5031..5634

/label=3'UTRintron

misc_feature complement(5612..5644)

/label=PUb rev

/note="PUb rev"

primer_bind complement(5612..5644)

/label=PUbRv-gttc

primer_bind 5645..5662

/label=EM692

CDS 5646..6362

/codon_start=1

/product="enhanced GFP"

/label=EGFP

/note="mammalian codon-optimized"

polyA_signal 6487..6568

/label=SV40 poly(A) signal

/note="SV40 polyadenylation signal"

primer_bind complement(6592..6605)

/label=EM1999

misc_feature complement(6623..6656)

/label=loxP

/note="loxP"

misc_feature 6670..6910

/label=piggyBac 3' sequence

/note="piggyBac 3' sequence"

misc_feature complement(7036..7052)

/label=M13F

/note="M13F"

ORIGIN

1 ctgccaggaa acagctatga ccatgtaata cgacgatatg atcctgatgc agctagatta

61 accctagaaa gatagtctgc gtaaaattga cgcatgcatt cttgaaatat tgctctctct

121 ttctaaatag cgcgaatccg tcgctgtgca tttaggacat ctcagtcgcc gcttggagct

181 cccgtgaggc gtgcttgtca atgcggtaag tgtcactgat tttgaactat aacgaccgcg

241 tgagtcaaaa tgacgcatga ttatctttta cgtgactttt aagatttaac tcatacgata

301 attatattgt tatttcatgt tctacttacg tgataactta ttatatatat attttcttgt

361 tatagattag atcgcgctcg cgcgactgac ggtcgtaagc acccgcgtac gtgtccaccc

421 cggtcacaac cccttgtgtc atgtcggcga ccctacgccc ccaactgaga gaactcaaag

481 gttaccccag ttggggcact actcccgaaa accgcttctg acctgggaaa acgtgaagcc

541 ccggggcatc cgctgagggt tgccgccggg gcttcggtgt gtccgtcagt actgcaggta

601 ccataacttc gtatagcata cattatacga agttataccg gatccatctt tacatgtagc

661 ttgtgcattg aatccaatta taatttgcct tggcaccagc tgagccagac aagaaagaaa

721 gcttcccaga agtatatcga tttagaaggg ttgacgtcac tttgctgact gcactaatac

781 agcaaatgat acaattagaa tgattcaagt gaaattccca aattactgct ttgtctctgg

841 atttggttat cagattacat tcgaagctaa gaatagctac cgaaattgtc gatcaaatca

901 ggaaatcctt tctctatcga aaaaggcatt cgcacatctt tctctgtatg ccatatacac

961 gaatggtagg tacattgacg tctttgccag aagttgaact gcatcgttca aggtacagaa

1021 tgaacgacta acagacacaa gcacgttttg ctgtccattc agacacaggg atggtaccca

1081 tagtcgatcg atttagagcc atccaaccga acagaggtat atgtatgaat ggattgcaga

1141 aattttctag aagtacaacc accactacgg cagtgtctat aaaacgcccc tgcaaaggca

1201 aaaccagctc aatcgaatac gtttcctagt ggagtgaaca ttacgcggtc caagtaagca

1261 gtgccagtgc aagtgaagtg aagtctctag tgaaaaagag tgatccaatt agccagagga

1321 gaaaatttca gagtgaacaa agctttgttc aaaggacaat tactattaaa tttgtgaaag

1381 tgcatttcgg tgaagggaat cttctagtga aggtaggtaa attaaatgat gaaattatag

1441 ctatgagcga aaactagttt ggtgaatgat tcctttgtct ttgaatgagc aaactatttt

1501 ccaagatggc gactattgag ctttgagtga ttagtgaaaa tttgcaacgc agtttcatca

1561 tcattgataa aacccaattg tgattcacgg cgataatcat atttcgttga atcatcgctg

1621 ctaattgaat taaatttcta gagcaagcgc gaattcgcca tatttctaaa attaaatatt

1681 gtggtgataa ttacccatta aggtaatatt aacacatatc gagaaaaacc ttgaggaaat

1741 cgtgaaaact tgaagatacg caatttccaa actacgtagt tcaaagtcga aaacaagtta

1801 atttttcact aaaaagtagg gcgttgttgt gacgtcatca ccttcaagtg tatatttttc

1861 acttggcctg cgactgcaaa cgcagacaaa gcaaaacaag tttaaaacct gtcgtgtcgt

1921 gctcgaagcc aaaggcaatg aatcaatatc aaatgagagt ttgcatttca caaccaatta

1981 ctgaagcgtt tcctcgtttc tttttctgct caacagagat ttcaacaatg agcgcggaaa

2041 ttgtggccga aaggggcagc agcctggtgt cgctggccgt gccgatggtc atttttatga

2101 ccctggtctt ggtagccagt gcattgttcc atttctggat gatatcccgg cggtacgttc

2161 aactgggaaa caagattccc ggaccgaggg cctatccgtt cattggcaat gccaatatgc

2221 tgctggggat gaaccacaat gagatcatgg agcgggcgat gcagttgagt tatatctatg

2281 gaagtgtggc tcggggctgg ctcgggtatc atttggtggt gtttttgacc gagccagcgg

2341 acattgagat catcctgaac agttatgtgc acctgacaaa gtccagcgag tacaggtttt

2401 tcaagccatg gcttggcgat gggctgttga tcagcagtgg cgagaagtgg cgatcacatc

2461 ggaagctaat cgctccggcg ttccatatga atgtcctgaa gacgttcgtg gatgtgttca

2521 acgataacag tttggcggtg gtggaacgga tgcgaaagga ggtggggaag gagttcgacg

2581 tgcacgacta tatgagtgaa gttacggtgg atattctgct ggagacggcc atgggatcac

2641 agaggacgag cgagagcaag gaaggattcg actatgcgat ggctgtgatg aaaatgtgtg

2701 acatcctaca ctcccgtcag ttgaaattcc acctccggat ggactccgtc ttcaacttca

2761 ccaaaatcaa gcaggaacag gaacgcctgc tcggcatcat ccacggcctc acccggaagg

2821 tcggcaagca gaagaaggaa ctcttcgaga agaatttcgc cgacggtaag ctgccttcgc

2881 cgtccctttc cgaaattatt gccaaggaag agtccgaatc caaagaaccg cttccggtca

2941 tctcgcaggg ttcgctcctc agggacgatc tggacttcaa cgatgaaaac gacatcggcg

3001 agaagcgaag gcttgccttc ctggacctga tgatcgaaac ggccaagagc ggtgccgatc

3061 tgaccgatga agagatcaag gaagaagtgg acaccatcat gtttgaagga cacgacacca

3121 ctgcggccgg atccagcttt gtgctgtgcc ttctcggcat tcaccaggac gttcaagatc

3181 gagtttacaa agaaatctac cagatctttg gcaactccaa gcggaaagct acattcaacg

3241 acaccttgga gatgaagtac ctggaacggg tgatctttga aaccttgaga atgtatccac

3301 cggttccggt gattgcccgc aaagtgaccc aagatgtccg gctggcttcc cacgactacg

3361 tggttccagc tggaaccacg gtcgtcatcg gtacttacaa agtgcaccga cgggcggaca

3421 tctaccctaa tccagatgtg ttcaacccgg acaatttcct accggaacgc acacagaatc

3481 gccactacta cagctacatc ccattcagcg ccggaccgcg aagttgcgtc ggtagaaaat

3541 atgccatgct gaaactgaag gtcctcctgt caaccatctt gcgcaactac agggtcgtgt

3601 ccaatctcaa ggaatcggac tttaagctac aaggcgacat tatcctgaaa cggaccgatg

3661 gcttcagaat acagctggaa ccgagagtct aatcaattat gaccacgaac tgagccaggc

3721 agggagccct cgggctgata agatgtcgcg acattgccga tggacaccga atggacgata

3781 gattaaatag aacttgttgt atagcctttt tatgatactg ttaattgaat tgttcggttc

3841 ccgattgcca ggtcgagccg attgttgttg cttttgcttt tgcttgatgc ttttgttttg

3901 cgcttcatca cagaacgtta tttatttgct gcttcgcttt cgctttctgt ccccttacga

3961 gcataaatgg gagataataa aaccttcttc gcttagatgc tagtgttgat ttttttcttc

4021 gtttcgagtt cattcatatc cgaaacaaaa ggattatcgt ctgaccccag acgacaacga

4081 tatcgcaaaa aagcggtcaa tatggtcaat caatgtttgt tgtcctgagc gtctcggaga

4141 gttgttaaga taagacggta agggtaagtc gatgaagttg ttccattaat gagtggtctt

4201 caagataagg gatacaatgt ttggttataa agcttcgcca aaacatgctt cctgggattg

4261 gggatcttta catgtagctt gtgcattgaa tccaattata atttgccttg gcaccagctg

4321 agccagacaa gaaagaaagc ttcccagaag tatatcgatt tagaagggtt gacgtcactt

4381 tgctgactgc actaatacag caaatgatac aattagaatg attcaagtga aattcccaaa

4441 ttactgcttt gtctctggat ttggttatca gattacattc gaagctaaga atagctaccg

4501 aaattgtcga tcaaatcagg aaatcctttc tctatcgaaa aaggcattcg cacatctttc

4561 tctgtatgcc atatacacga atggtaggta cattgacgtc tttgccagaa gttgaactgc

4621 atcgttcaag gtacagaatg aacgactaac agacacaagc acgttttgct gtccattcag

4681 acacagggat ggtacccata gtcgatcgat ttagagccat ccaaccgaac agaggtatat

4741 gtatgaatgg attgcagaaa ttttctagaa gtacaaccac cactacggca gtgtctataa

4801 aacgcccctg caaaggcaaa accagctcaa tcgaatacgt ttcctagtgg agtgaacatt

4861 acgcggtcca agtaagcagt gccagtgcaa gtgaagtgaa gtctctagtg aaaaagagtg

4921 atccaattag ccagaggaga aaatttcaga gtgaacaaag ctttgttcaa aggacaatta

4981 ctattaaatt tgtgaaagtg catttcggtg aagggaatct tctagtgaag gtaggtaaat

5041 taaatgatga aattatagct atgagcgaaa actagtttgg tgaatgattc ctttgtcttt

5101 gaatgagcaa actattttcc aagatggcga ctattgagct ttgagtgatt agtgaaaatt

5161 tgcaacgcag tttcatcatc attgataaaa cccaattgtg attcacggcg ataatcatat

5221 ttcgttgaat catcgctgct aattgaatta aatttctaga gcaagcgcga attcgccata

5281 tttctaaaat taaatattgt ggtgataatt acccattaag gtaatattaa cacatatcga

5341 gaaaaacctt gaggaaatcg tgaaaacttg aagatacgca atttccaaac tacgtagttc

5401 aaagtcgaaa acaagttaat ttttcactaa aaagtagggc gttgttgtga cgtcatcacc

5461 ttcaagtgta tatttttcac ttggcctgcg actgcaaacg cagacaaagc aaaacaagtt

5521 taaaacctgt cgtgtcgtgc tcgaagccaa aggcaatgaa tcaatatcaa atgagagttt

5581 gcatttcaca accaattact gaagcgtttc ctcgtttctt tttctgctca acagagattt

5641 caacaatggt gagcaagggc gaggagctgt tcaccggggt ggtgcccatc ctggtcgagc

5701 tggacggcga cgtaaacggc cacaagttca gcgtgtccgg cgagggcgag ggcgatgcca

5761 cctacggcaa gctgaccctg aagttcatct gcaccaccgg caagctgccc gtgccctggc

5821 ccaccctcgt gaccaccctg acctacggcg tgcagtgctt cagccgctac cccgaccaca

5881 tgaagcagca cgacttcttc aagtccgcca tgcccgaagg ctacgtccag gagcgcacca

5941 tcttcttcaa ggacgacggc aactacaaga cccgcgccga ggtgaagttc gagggcgaca

6001 ccctggtgaa ccgcatcgag ctgaagggca tcgacttcaa ggaggacggc aacatcctgg

6061 ggcacaagct ggagtacaac tacaacagcc acaacgtcta tatcatggcc gacaagcaga

6121 agaacggcat caaggtgaac ttcaagatcc gccacaacat cgaggacggc agcgtgcagc

6181 tcgccgacca ctaccagcag aacaccccca tcggcgacgg ccccgtgctg ctgcccgaca

6241 accactacct gagcacccag tccgccctga gcaaagaccc caacgagaag cgcgatcaca

6301 tggtcctgct ggagttcgtg accgccgccg ggatcactct cggcatggac gagctgtaca

6361 agtaaagcgg ccgcgactct agatcaaatc agccatacca catttgtaga ggttttactt

6421 gctttaaaaa acctcccaca cctccccctg aacctgaaac ataaaatgaa tgcaattgtt

6481 gttgttaact tgtttattgc agcttataat ggttacaaat aaagcaatag catcacaaat

6541 ttcacaaata aagcattttt ttcactgcat tctagttgtg gtttgtccaa actcatcaat

6601 gtatcgcttg taattcgtcg acataacttc gtatagcata cattatacga agttatgagc

6661 tcaattcgat aaaagttttg ttactttata gaagaaattt tgagtttttg ttttttttaa

6721 taaataaata aacataaata aattgtttgt tgaatttatt attagtatgt aagtgtaaat

6781 ataataaaac ttaatatcta ttcaaattaa taaataaacc tcgatataca gaccgataaa

6841 acacatgcgt caattttacg catgattatc tttaacgtac gtcacaatat gattatcttt

6901 ctagggttaa tctagctgcg tgttctgcag cgtgtcgagc atcttcatct gctccatcac

6961 gctgtaaaac acatttgcac cgcgagtctg cccgtcctcc acgggttcaa aaacgtgaat

7021 gaacgaggcg cgctcactgg ccgtcgtttt acaggggatg tcttcatata tatgaagact

7081 cccatctgtt gtttgtcggt gaacgctctc ctgagtagga caaatccgcc gggagcggat

7141 ttgaacgttg tgaagcaacg gcccggaggg tggcgggcag gacgcccgcc ataaactgcc

7201 aggcatcaaa ctaagcagaa ggccatcctg acggatggcc tttttgcgtt tctacaaact

7261 cttcctggct agcggtacgc gtattaattg cgttgcgctc actgcccgct ttccagtcgg

7321 gaaacctgtc gtgccagctg cattaatgaa tcggccaacg cgcggggaga ggcggtttgc

7381 gtattgggcg ctcttccgct tcctcgctca ctgactcgct gcgctcggtc gttcggctgc

7441 ggcgagcggt atcagctcac tcaaaggcgg taatacggtt atccacagaa tcaggggata

7501 acgcaggaaa gaacatgtga gcaaaaggcc agcaaaaggc caggaaccgt aaaaaggccg

7561 cgttgctggc gtttttccat aggctccgcc cccctgacga gcatcacaaa aatcgacgct

7621 caagtcagag gtggcgaaac ccgacaggac tataaagata ccaggcgttt ccccctggaa

7681 gctccctcgt gcgctctcct gttccgaccc tgccgcttac cggatacctg tccgcctttc

7741 tcccttcggg aagcgtggcg ctttctcata gctcacgctg taggtatctc agttcggtgt

7801 aggtcgttcg ctccaagctg ggctgtgtgc acgaaccccc cgttcagccc gaccgctgcg

7861 ccttatccgg taactatcgt cttgagtcca acccggtaag acacgactta tcgccactgg

7921 cagcagccac tggtaacagg attagcagag cgaggtatgt aggcggtgct acagagttct

7981 tgaagtggtg gcctaactac ggctacacta gaagaacagt atttggtatc tgcgctctgc

8041 tgaagccagt taccttcgga aaaagagttg gtagctcttg atccggcaaa caaaccaccg

8101 ctggtagcgg tggttttttt gtttgcaagc agcagattac gcgcagaaaa aaaggatctc

8161 aagaagatcc tttgatcttt tctacggggt ctgacgctca gtggaacgac gcgtaactca

8221 cgttaaggga ttttggtcat gagcttgcgc cgtcccgtca agtcagcgta atgctctgcc

8281 agtgttacaa ccaattaacc aattctgatt agaaaaactc atcgagcatc aaatgaaact

8341 gcaatttatt catatcagga ttatcaatac catatttttg aaaaagccgt ttctgtaatg

8401 aaggagaaaa ctcaccgagg cagttccata ggatggcaag atcctggtat cggtctgcga

8461 ttccgactcg tccaacatca atacaaccta ttaatttccc ctcgtcaaaa ataaggttat

8521 caagtgagaa atcaccatga gtgacgactg aatccggtga gaatggcaaa agtttatgca

8581 tttctttcca gacttgttca acaggccagc cattacgctc gtcatcaaaa tcactcgcat

8641 caaccaaacc gttattcatt cgtgattgcg cctgagcgag acgaaatacg cgatcgctgt

8701 taaaaggaca attacaaaca ggaatcgaat gcaaccggcg caggaacact gccagcgcat

8761 caacaatatt ttcacctgaa tcaggatatt cttctaatac ctggaatgct gtttttccgg

8821 ggatcgcagt ggtgagtaac catgcatcat caggagtacg gataaaatgc ttgatggtcg

8881 gaagaggcat aaattccgtc agccagttta gtctgaccat ctcatctgta acatcattgg

8941 caacgctacc tttgccatgt ttcagaaaca actctggcgc atcgggcttc ccatacaagc

9001 gatagattgt cgcacctgat tgcccgacat tatcgcgagc ccatttatac ccatataaat

9061 cagcatccat gttggaattt aatcgcggcc tcgacgtttc ccgttgaata tggctcataa

9121 caccccttgt attactgttt atgtaagcag acagttttat tgttcatgat gatatatttt

9181 tatcttgtgc aatgtaacat cagagatttt gagacacggg ccagag

//
